# Completion surgery after intensity-modulated arc therapy for locally advanced cervical cancer: long-term follow-up and update on surgical outcome and oncologic results of a unique tertiary care single-center retrospective cohort

**DOI:** 10.1186/s12957-023-02971-5

**Published:** 2023-03-08

**Authors:** Axel Van Damme, Julie Rombaut, Amin Makar, Emiel De Jaeghere, Eline Naert, Hannelore Denys, Rawand Salihi, Philippe Tummers, Katrien Vandecasteele

**Affiliations:** 1 Radiation Oncology, Ghent University Hospital, Ghent, Belgium; 2 Department of Gynaecology, Division of Gynecologic Oncology, ZNA Middelheim, Antwerp, Belgium; 3 Gynecological Pelvic Oncology Network (GYPON), Ghent University Hospital, Ghent, Belgium; 4 Department of Gynaecology, Ghent University Hospital, Ghent, Belgium; 5 Medical Oncology, Ghent University Hospital, Ghent, Belgium; 6 Laboratory of Experimental Cancer Research (LECR), Ghent University, Ghent, Belgium; 7 Cancer Research Institute Ghent (CRIG), Ghent University, Ghent, Belgium; 8 Department of Gynaecology, AZ Sint-Lucas, Ghent, Belgium

**Keywords:** Cervical cancer, Radiotherapy, Surgical oncology

## Abstract

**Background:**

Chemoradiotherapy (CRT) followed by brachytherapy (BT) is the standard treatment for locally advanced cervical cancer (LACC), but replacement of BT by surgery (CRT-S) could be an acceptable alternative. The main concern is the risk of operative morbidity. The aim is to report on therapeutic morbidity, OS, PC, and LC of CRT-S.

**Methods:**

This was a single tertiary center retrospective cohort study in patients treated with CRT-S. A type II Wertheim hysterectomy was performed 6–8 weeks after CRT. Acute and chronic radiotherapy-related and surgical morbidity was classified according to the CTCAE v4.0. OS, and DFS, PC, and LC were calculated using the Kaplan-Meier method. Univariate and multivariate Cox proportional hazard models were performed to determine variables with a prognostic role.

**Results:**

A total of 130 consecutive LACC patients were treated with CRT, and 119 underwent completion surgery. The median follow-up was 53 months. Five-year OS rate, local control, pelvic control, and 5-year DFS rate were 73%, 93%, 90%, and 74%, respectively. The 5-year OS rate was 92%/72%/67%/56% for FIGO (2009) stage I/II/III/IV, respectively. The five-year OS rate was 79% and 71% for adenocarcinoma and squamous cell carcinoma (*p* > 0.05), respectively. There was no intra- and perioperative mortality. Intraoperative and early postoperative complication rates were 7% and 20% (3% ≥ G3), respectively; they resolved within 3 months. The late postoperative complication rate was 9% (7% ≥ G3). Acute/late radiotherapy-related G3 side effects were 5%/3% for gastrointestinal and 3%/7% for genitourinary side effects.

**Conclusions:**

CRT-S is safe with an acceptable rate of complications for both the CRT and completion surgery and shows encouraging outcome data for stage III/IV and adenocarcinoma patients.

**Supplementary Information:**

The online version contains supplementary material available at 10.1186/s12957-023-02971-5.

## Background

Cancer of the uterine cervix is the fourth most common cancer worldwide and the fourth leading cause of cancer deaths in females [1]. Yearly, over 58,000 patients are diagnosed with and around 25,000 patients die of cervical cancer in Europe. The 5-year relative survival for European cervical cancer patients in 2000–2007 was 63% [2].

Management of patients with cervical cancer mainly depends on the stage of disease at diagnosis and histology. The International Federation of Gynecology and Obstetrics (FIGO) stage IB2-IVA (FIGO 2009) or stage IB3–IVA (FIGO 2018) can be defined as locally advanced cervical cancer (LACC). The standard treatment for LACC is definitive chemoradiation (CRT-BT), consisting of concurrent chemoradiotherapy (CRT) followed by a (image-guided adaptive) brachytherapeutic boost (BT) [3–5]. Also, some selected patients with stage IVB (e.g., oligometastatic disease or supraclavicular lymph nodes) benefit from definitive chemoradiotherapy [6–9].

An alternative but controversial approach to LACC is CRT followed by completion surgery (S) (CRT-S). Because the extent of residual disease is directly related to the risk of relapse, completion surgery could lead to a reduced recurrence rate and an improved prognosis [10, 11]. In addition, the completion surgery enables the evaluation of the pathologic response [12]. The main concern of completion surgery is the potentially higher morbidity by operating on an irradiated pelvis [10]. Since the development of intensity-modulated radiotherapy (IMRT), radiation therapy has evolved from a nontargeted approach to a precisely targeted, highly conformal treatment. The positive impact on healthy surrounding tissues and morbidity has been proven [13]. The current guidelines advise systemic radical hysterectomy after CRT-BT in non-metastasized patients with residual tumor (≥ 6- to 8-week assessment), confirmed histologically or by serial radiological follow-up [7].

Until now, three phase 3 randomized trials have been published on CRT-S. Keys et al. compared 124 patients receiving CRT-B with 132 patients receiving CRT-S [14]. They found a 5-year DFS rate of 62% after hysterectomy compared to 53% without surgery for stage IB2 cervical cancer (*P* = 0.09) with a significant difference when comparisons were adjusted for tumor size, performance status, and age (*P* = 0.04). Cetina et al. compared chemoradiotherapy and brachytherapy with chemoradiotherapy followed by type III radical hysterectomy [15]. The 211 enrolled patients (100 CRT-B, 111 CRT-S) received 50.4 Gy combined with six courses of cisplatin 40 mg/m^2^ and gemcitabine 125 mg/m^2^. No difference in OS, PFS, local failure, and systemic failure could be demonstrated; therefore, the study concluded CRT-S was not superior to the standard of care CRT-B. Morice et al. compared 61 patients with stage IB2 or stage II cervical cancer without extrapelvic disease on conventional imaging who received 45 Gy with or without parametrial or nodal boost and concomitant Cisplatin 40 mg/m^2^ weekly. Of the 61 patients, 31 were randomized to CRT-S and 30 to CRT-B. Both the 3-year OS as event-free survival were equal in both arms. There is no general consensus in the literature on the benefit of the use of CRT-S [16, 17].

We previously reported that due to the implementation of advanced radiotherapy techniques with a higher radiation dose on the target volume and a lower dose to the organs at risk and surrounding tissues, we created the opportunity to safely perform a radical hysterectomy and a tailored lymphadenectomy [18]. This report is an update to our 2013 publication and presents the long-term survival, local control (LC), pelvic control (PC), and acute and long-term surgical- and radiotherapy-related morbidity of this multimodality treatment. We also investigated the impact of histology and the degree of pathological response on outcome.

## Methods

### Patients

We retrospectively reviewed patients with biopsy-proven LACC treated from 2005 to 2020 [19]. This retrospective study was approved by the local ethics committee (UZ Gent 2019/1089). Informed consent was obtained from all individual participants included in the study. The findings have been reported according to the STROBE guidelines.

Clinical staging (FIGO, both 2009 and 2018) at diagnosis was obtained by pelvic examination by an experienced gynecologic oncologist and a radiation oncologist. In addition, all patients were staged by total-body 18FDG PET-CT and pelvic magnetic resonance imaging (MRI) and staged by TNM 8 [20]. Patients were considered node positive when nodes were 18FDG-positive or had a minimal diameter of 1 cm (oval lymph nodes) or 8 mm (round lymph nodes) when 18FDG-negative.

### Treatment and follow-up

The eligibility criteria are biopsy-proven locally advanced (FIGO IB2-IVA) cervical cancer (LACC), absence of distant metastases, and extrapelvic lymph node(s) as diagnosed on fluorine 18 fludeoxyglucose (18FDG) positron emission tomography-computed tomography (PET-CT), World Health Organization scores 0–2, and ability to understand and sign informed consent [18]. Patients underwent neo-adjuvant intensity-modulated arc therapy (VMAT), combined with weekly cisplatin (40 mg/m^2^) or 5-fluorouracil in case of inadequate kidney function. Details concerning the delineation, dose description, planning, and delivery of VMAT were previously reported [21]. In short, a median dose (D_50_) of 62 Gy and a minimal dose (D98) of 45 Gy were delivered to the planning target volume (PTV) of the primary tumor (GTV) and PTV of the non-involved uterus, parametria, and upper 1/3 of the vagina. In addition, a D_50_ of 60 Gy was delivered to the PTV of positive lymph nodes, and a D_98_ of 45 Gy was delivered to the PTV of the elective lymph nodes. As per institutional protocol, 4 weeks after completing CRT, the possibility to perform adjuvant surgery was evaluated based on imaging (18FDG PET-CT and MRI, both performed at 3 weeks post-CRT) and gynecologic examination [22, 23].

All surgical procedures were attempted in patients achieving clinical response to CRT or stable disease. A radical hysterectomy was performed 6–8 weeks after the completion of CRT. Surgery consisted of a type II Wertheim hysterectomy. Although the LACC trial did not include patients after chemotherapy and radiation, we chose to no longer expose our patients to the possible risks of minimally invasive surgery and abandoned robotic surgery after publication [24].

Pelvic lymphadenectomy was performed whenever there were suspicious lymph nodes present on the 18FDG PET-CT. From August 2008 onward, lymph node dissection was limited to the lymph node regions that were positive on one of the 18FDG PET-CTs (elective lymphadenectomy).

We performed both open and robotic-assisted surgery. From 2018 onward, however, patients were no longer offered robotic-assisted surgery due to the results of the LACC trial [24].

Follow-up was scheduled 3-monthly (years 1–2), 6-monthly (years 3–5), and yearly thereafter at a multidisciplinary consultation. Toxicity and pelvic and distant control were evaluated at every visit by gynecologic and general clinical examination. Imaging (18FDG PET-CT and MRI) was performed every 6 months for the first 2 years and yearly thereafter, or when symptoms were present.

### Key definitions

DFS was defined as the time from the initial diagnosis (histology) to disease recurrence or death from any cause. OS was defined as the time from initial diagnosis (histology) to death from any cause. PC is defined as the absence of local and nodal disease within the pelvis. LC was defined as the absence of disease in postoperative hysterectomy region, upper vagina, and parametria on gynecologic examination at follow-up. Data regarding patients with no evidence of recurrence or death were censored at the date of the last follow-up. Follow-up was defined as the time from the end of treatment to the relevant event (death from any cause, cancer-specific death, any recurrence, local recurrence, and pelvic recurrence).

All toxicity data were scored using the Common Terminology Criteria of Adverse Events (CTCAE) version 4.0.

Gastrointestinal (GI) and genitourinary (GU) radiotherapy-related toxicities were categorized into acute (symptoms experienced during or ≤ 3 months of completion of CRT-S) and chronic (> 3 months after CRT-S).

Surgical morbidity and mortality were evaluated and registered during hospitalization and postoperative (acute, ≤ 6 weeks postoperative) and at every visit thereafter (late). Based on CTCAE v4, the following data were extracted: urinary infection, wound infection, urinary fistula, digestive fistula, ileus, bowel subobstruction, and thromboembolic events.

Pathology results were analyzed with regard to resection margins and pathological response (residual tumor was defined as ≥ 10 mm grossly and < 10 mm microscopically); they were also compared with the imaging performed after CRT.

### Statistical analysis

Descriptive analyses were performed for demographic, clinicopathologic, and treatment data. Survival curves for time-to-event endpoints and cumulative survival rates were estimated using the Kaplan-Meier method. The log-rank test was used to compare the groups. Missing data were not imputed. All reported *P* values are 2-tailed with significance levels at *P* ≤ 0.05 with no adjustments for multiplicity. Data analysis and visualization were performed using SPSS version 25 (IBM Corporation, Armonk, NY, USA) and R version 4.0.1 (R Foundation for Statistical Computing, Vienna, Austria). The data cutoff for the analysis was January 25, 2021. Data analysis was conducted from April 24, 2021, to July 26, 2021. We used univariate and multivariate Cox proportional hazard models to select the variables with a prognostic role in the whole series. The variables considered in the logistic regression model were preoperative parameters and were chosen for their clinical relevance according to the investigators’ opinion. Variables with *P* < 0.05 at univariate analysis were included in the multivariate analysis.

## Results

### Patient characteristics

Between August 2005 and February 2020, 130 consecutive patients with LACC were included: the intention-to-treat or ITT group. Ten patients who did not undergo surgery and one patient who only received lymphadenectomy after CRT were excluded; 119 patients underwent surgery (CRT-S group). Indications for not undergoing surgery were progressive disease (*n* = 2), insufficient tumor response (defined as tumor shrinkage < 50%) (*n* = 7), poor general condition (*n* = 1), and refusal of surgery (*n* = 1). The median follow-up was 53 months.

Patient characteristics are summarized in Table 1.

**Table 1:** Patient characteristics

	Intention-to-treat	CRT-S
**Number of patients,** ***n***	130	119
**Age at diagnosis, mean (range)**	55 (25–82)	53 (25–80)
**Follow-up in months, mean (range)**	48 (4.73–170.57)	53 (7.30–170.57)
**Histology**		
**Adenocarcinoma**	23 (18%)	23 (19%)
**Squamous cell carcinoma**	107 (82%)	97 (81%)
**Tumor size in cm, mean (range)**	5.1 (1.5–11.7)	4.7 (1.5–11.7)
**FIGO 2009**		
**I**	12 (9%)	12 (10%)
**II**	85 (65%)	80 (67%)
**III**	22 (17%)	19 (16%)
**IV**	11 (9%)	8 (7%)
**FIGO 2018**		
**IB3**	4 (3%)	4 (3%)
**II**	49 (38%)	46 (39%)
**III**	64 (49%)	59 (50%)
**IV**	13 (10%)	10 (8%)
**Chemotherapy**	120 (92%)	112 (94%)
**Positive lymph nodes**	66 (51%)	63 (53%)
**Iliac artery**	57	54
**Inguinal**	2	2
**Para-aortic**	7	7
**Clinical complete response,** ***n*** **(%)**	43 (33%)	43 (36%)
**Iconographic complete response,** ***n*** **(%)**	59 (45%)	56 (47%)
**Cancer-related deaths,** ***n*** **(%)**	30 (23%)	22 (18%)

Iconographic complete response was measured using MRI/18FDG-PET CT, and tumor size was measured as the largest diameter on MRI

### Outcomes of CRT-S

**Fig. 1:**
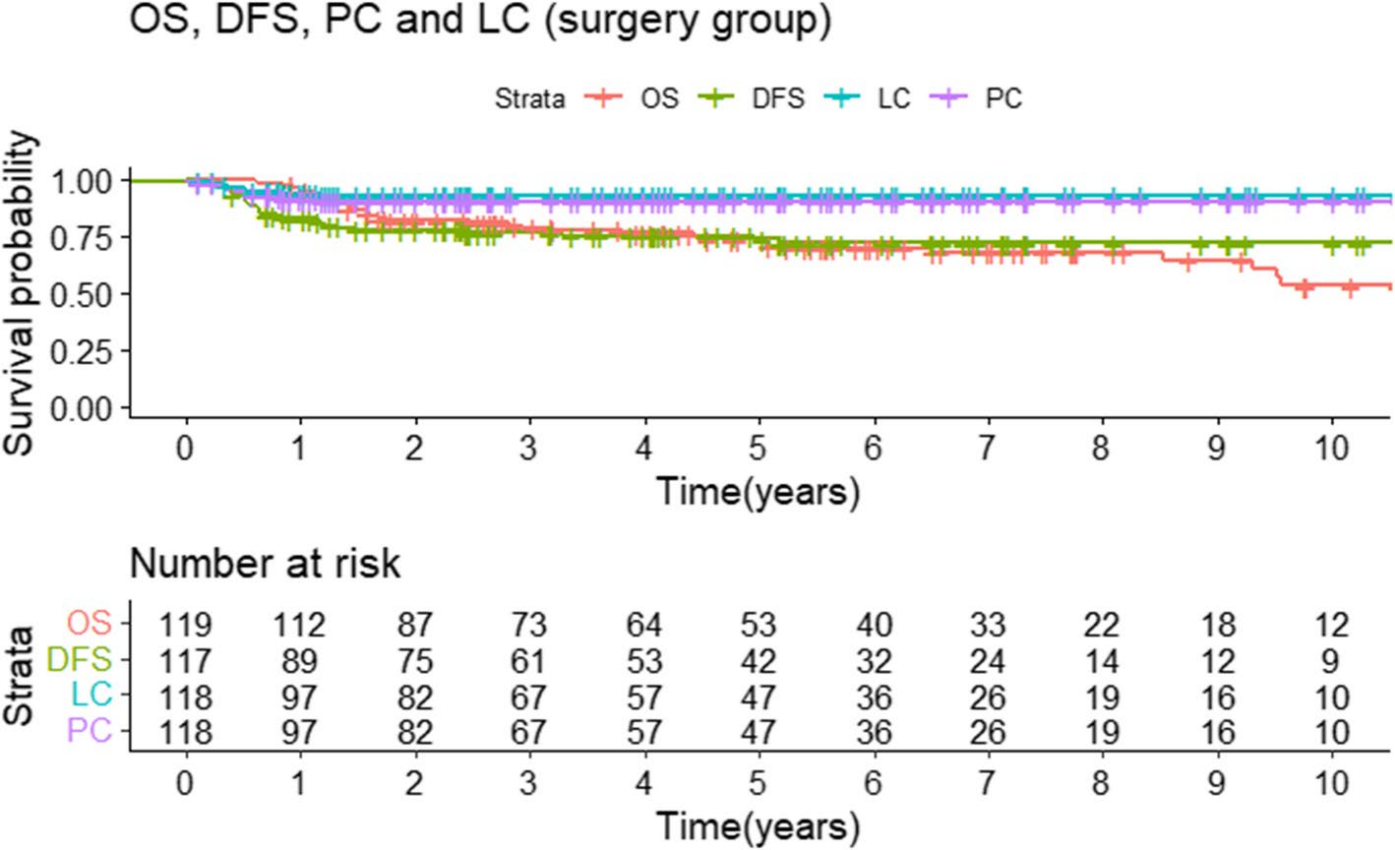
Overall survival (OS), disease-free survival (DFS), pelvic control (PC), and local control (LC) in the CRT-S

Data for the ITT group and confidence intervals of the below-mentioned survival analyses can be found in Additional file 1: Table S1, and Fig. 1.

#### Mortality

Thirty-six patients died. Twenty-two patients died of tumor progression, and 2 patients chose euthanasia. For 7 patients, the cause of out-of-hospital death was unknown of which 2 patients likely died due to tumor progression (progressive and in follow-up shortly before death). Two patients died due to a secondary tumor (lung cancer and adenocarcinoma of the colon), and 2 and 1 patients died from kidney failure and infective disease, respectively.

#### Overall survival

**Fig. 2:**
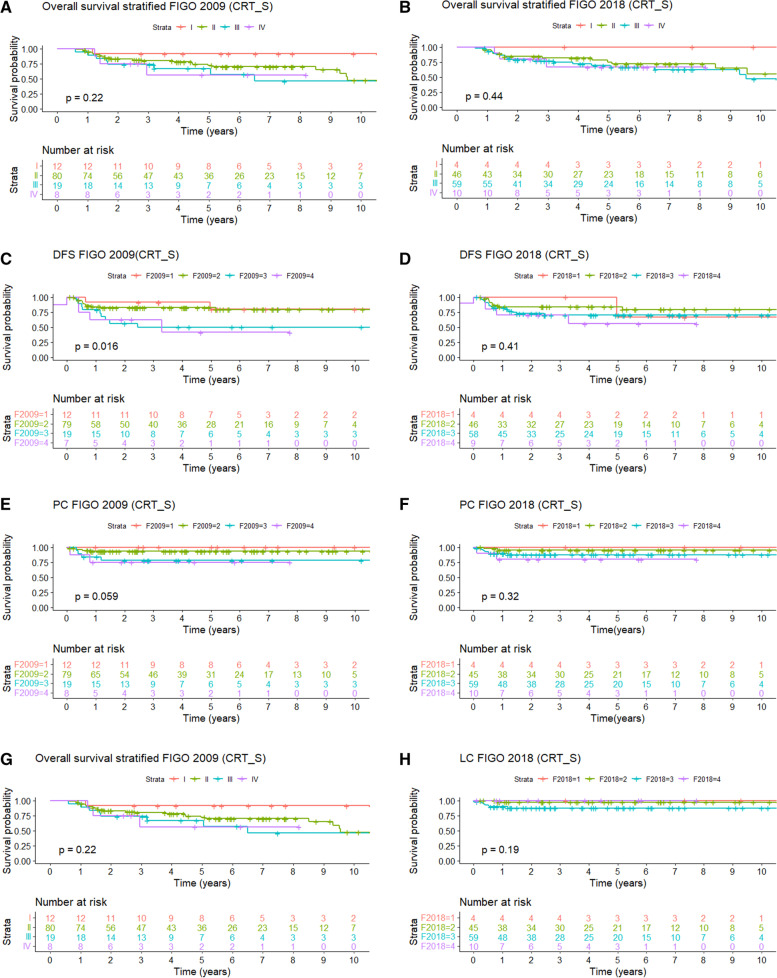
OS, DFS, PC, and LC stratified according to FIGO 2009 (respectively, **A**, **C**, **E**, and **G**) and 2018 (respectively, **B**, **D**, **F**, and **H**) in the CRT-S

Five- and 10-year OS are 73% and 53%, respectively (Fig. 1). The 5-year OS was 92%, 72%, 67%, and 56% for FIGO 2009 stages I, II, III, and IV, respectively. Using FIGO 2018, this was 100%, 75%, 69%, and 67%, respectively (see Fig. 2).

The 5-year OS was not statistically significantly different (*P*-value = 0.17 and 0.56) for AC (79%) compared to SCC (71%) or N1 (70%) compared to N0 (75%) status.

#### Disease-free survival

Five-year and 10-year DFS is 74% and 72% (Fig. 1). Staged according to FIGO 2009, 5-year DFS was 80%, 79%, 46%, and 30% for stages I, II, III, and IV, respectively. Using FIGO 2018, this was 67%, 81%, 66%, and 43%, respectively (see Fig. 2).

Five-year DFS was not statistically significant (*P* = 0.31) different for AC (61%) compared to SCC (73%) or N1 (75%) compared to N0 (74%) status.

#### Local and pelvic control

The 5-year and 10-year LC were 93% (Fig. 1) and did not differ significantly (*P* = 0.62) between AC/SCC: 95%/92%. When we staged according to FIGO 2009, 5-year LC was 100%%, 95%, 78%, and 100% for stages I, II, III, and IV, respectively. Stratified by FIGO 2018 stage for the surgery group, 5-year LC was 100%, 98%, 88%, and 100% for stage I, stage II, stage III, and stage IV, respectively (see Fig. 2).

Five-year PC was 90% and did not differ significantly (*P* = 0.35) when stratified according to lymph node status or histology at diagnosis: 88%/91% for N1/N0 status and 89%/95% for SCC/ACC.

#### Univariate and multivariate analysis

In the univariate analysis of the pre-treatment features potentially associated with OS, only age at the time of diagnosis was associated with an increased risk of death. No multivariate analysis was performed, because only one factor was significant in univariate analysis.

In the univariate analysis of the pre-treatment features potentially associated with DFS, none was associated with an increased risk of recurrence. Because no factors were significant, no multivariate analysis was performed.

### Toxicity

#### Radiotherapy-related toxicity

Chronic toxicity had 25 missings due to loss to follow-up (*n* = 12) or death (*n* = 13). These patients were excluded only in chronic toxicity reporting.

Acute and chronic GI toxicity (all grades) was seen in 91% and 46% of patients, of which 5% and 3% were ≥ G3, respectively. Acute and chronic GU toxicity (all grades) was seen in 67% and 40%, of which 3% and 7% were G3, respectively. No G4 side effects were observed. Acute G3 GI toxicity was seen in six patients: five were hospitalized for nausea, vomiting, and dehydration, one patient needed intravenous analgesics for abdominal pain. Chronic G3 GI toxicity was bowel (sub)obstruction in all three patients. Acute G3 toxicity consisted of urinary infections. Chronic G3 GU toxicity was seen in seven patients: six were treated with transobturator tape for urinary incontinence, and one patient was hospitalized with a urinary infection. Table 2 gives an overview of the data.

**Table 2:** Maximal gastrointestinal and urinary radiation-related toxicity in the CRTs group according to CTCAE v4

CTCAE v5 grade	Gastrointestinal		Urinary	
	Acute, ***N*** = 119	Chronic, ***N*** = 94	Acute, ***N*** = 119	Chronic, ***N*** = 94
0	11 (9%)	51 (54%)	39 (33%)	56 (60%)
1	10 (8%)	24 (26%)	41 (34%)	19 (20%)
2	92 (77%)	16 (17%)	36 (30%)	12 (13%)
3	6 (5%)	3 (3%)	3 (3%)	7 (7%)
4	0	0	0	0
5	0	0	0	0

#### Surgery-related toxicity

Intraoperative injuries occurred in 7 patients (6%) and included bladder injury (G1, *n* = 3), serosal bowel injury (G1, *n* = 3), and femoral neuropathy (G2, *n* = 1). Eleven patients (9%) required a postoperative blood transfusion. Six patients (5%) had an estimated blood loss of > 1 l.

The operative mortality was nil. Twenty-eight patients (23%) experienced any grade postoperative complications. Grade 1–2 complications occurred in 22 patients (18%), and G3-4 complications occurred in 14 patients (12%).

Tables 3 and 4 summarize all early and late postoperative complications, respectively, according to organ system and grade. One patient needed a postoperative re-intervention (day 5), due to an acute abdomen with suspicion of intestinal ischemia (not confirmed, only inflammation of the intestine with caliber changes was seen).

**Table 3:** Type of early postoperative complications in the CRTs group according to organ system and grade according to CTCAE v4

Organ system	***N*** (%)
Experienced postoperative complications	24 (20%)
Urinary infection	4 (3%)
Grade 2	3 (3%)
Grade 3	1 (1%)
Urinary retention	15 (13%)
Grade 1	2 (2%)
Grade 2	13 (11%)
Ileus	2 (2%)
Grade 2	1 (1%)
Grade 3	1 (1%)
Subobstruction	1 (1%)
Grade 4	1 (1%)
Pelvic infection	1 (1%)
Grade 3	1 (1%)
Thromboembolic event	1 (1%)
Grade 2	1 (1%)

**Table 4:** Type of late postoperative complications according to organ system and grade according to CTCAE v4

Organ system	***N*** (%)
All	11/119 (9%)
Urinary fistulae	1 (1%)
Grade 3	1 (1%)
Ileus	3 (3%)
Grade 3	3 (3%)
Bowel subobstruction	3 (3%)
Grade 3	2 (2%)
Grade 4	1 (1%)
Pelvic infection	2 (2%)
Grade 3	2 (2%)
Wound infection	2 (2%)
Grade 1	2 (2%)

Fifteen patients (13%) had problems with urinary retention when the bladder catheter was removed. At the time of discharge, 13 patients required self-catheterization, but all urinary retention problems resolved spontaneously (< 3 months). Two patients developed an ileus, and both cases were managed conservatively. One patient needed a postoperative re-intervention (day 5), due to an acute abdomen with suspicion of intestinal ischemia (not confirmed, only inflammation of the intestine with caliber changes was seen). One patient developed a deep venous thrombosis 4 weeks after surgery. No urinary or digestive fistula, urinary stenosis, hemorrhage, wound infection, or pulmonary embolism was seen.

Thirty-three patients received robotic surgery. Blood loss was minimal and only one of the patients (3%) needed a postoperative transfusion. No urinary or bowel injury was seen. One patient (3%) had a grade 2 pelvic infection. Three patients (9.1%) experienced postoperative urinary retention. There was no observed postoperative urinary or digestive fistula. One patient had a wound infection. No thromboembolic events were recorded. No postoperative re-interventions were necessary.

### Pathology

Tumor resection margins were free of disease (R0) and narrow (< 1 mm) in all but four and one cases, respectively, and those patients received adjuvant BT. Of interest, nine patients (90%) with T4 tumor had a complete resection. The complete pathological response rate was 41%. Of the 70 patients with residual disease (RD), 31 (26%) had grossly and 39 (33%) had microscopic RD. Residual tumor was present in the pelvic lymph nodes in 19% of patients with positive lymph nodes on pretreatment FDG PET-CT. Of the 59 patients who had a complete clinical response on the evaluation MRI performed after CRT, 26 patients (44%) had pathologic RD. Of the 60 patients who seemed to have RD on MRI, 16 patients (27%) showed complete responses. Nineteen patients out of 23 patients (83%) with adenocarcinoma had RD on MRI.

## Discussion

Completion of hysterectomy after CRT is still under debate due to unclear survival benefits and potentially increased morbidity [11, 25, 26]. We report on a single tertiary center experience concerning 119 patients with LACC treated with CRT and completion hysterectomy, an update on an earlier published cohort [18]. In this cohort, 5-year OS, LC, PC, and DFS for the patients receiving a hysterectomy were 73%, 92%, 90%, and 74 %, respectively. Stratified according to FIGO (2009), 5-year OS was 92%, 72%, 67%, and 56% for stages I, II, III, and IV, respectively. The large cohort of 731 patients with LACC treated with CRT-BT of the retroEMBRACE (IntErnational MRI-guided BRAchytherapy in CErvical cancer) study shows a 5-year OS of 65%, stratified according to FIGO 2009 stage IB 83%, 70% IIB, and 42% IIIB [27]. Furthermore, the 5-year overall PC in the retroEMBRACE data was 84% [28]. In the 26th FIGO annual report, the 5-year OS for patients with locally advanced cervical cancer (LACC) ranges from 66% for patients with stage IIB, 40% for stage III, and 22% for stage IVA. Salvage surgery could possibly benefit FIGO stage III–IV cervical cancer, but future research with head-to-head comparison is necessary.

According to a 2016 French survey, one-third of academic centers in France still perform completion hysterectomy in patients with complete response to CRT and negative para-aortic lymph nodes [29]. A recent systematic review and updated meta-analysis, based on retrospective studies, showed improved OS, increased DFS, and lower recurrence for patients receiving CRT-S [17]. However, data from small and controversial RCT showed no significant benefit in OS or DFS of adding completion surgery to SOC. Importantly, they only included stage IB2 or II cervical cancer [14, 15, 30]. No RCTs were performed in stage III–IV LACC.

The main concern of completion hysterectomy is the risk of complications while operating on an irradiated pelvis. The introduction of IMRT significantly lowered ≥ G3 radiotherapy-related toxicity [13, 31]. In addition, IMRT reduces the dose to the supportive tissues, making complementary surgery easier, certainly from a technical point of view. This also enables performing a radical hysterectomy (type II Wertheim) to remove the parametria and a vaginal manchet of approximately 2 cm. In our population, even though 58% of patients (*n* = 69) were FIGO stage III or IV, we achieved an R0 resection rate of 97%. Previous series in non-developed countries reported increased morbidity and mortality (1 out of 40 patients) [32].

We report no > G3 radiotherapy-related side effects. We reported 5%/3% acute/chronic G3 GI toxicity and 2.5%/7% acute/chronic G3 GU side effects. Chronic grade 3–5 side effects reported in the retroEMBRACE data were 6.8% for the bladder and 8.5% for the GI tract [33]. However, a recent update of EMBRACE-I showed very low late grade ≥ 3 GI toxicity of 2.8%, 1.8%, and 2.3% for anus/rectum, sigmoid, and colon/small bowel events, respectively [34]. Other studies showed that grade 3–4 toxicity in LACC treated with CRT-BT was between 8 and 11% [35–37].

Sexual health after both radiotherapy and surgery remains an important issue, and exploring techniques to preserve sexual function should be explored further (e.g., nerve-sparing radical hysterectomy) [38]. In addition, not only medical but also psychological and social factors are responsible for decreased sexual health of cervical cancer survivors [39, 40]. Therefore, discussing this topic timely with the patient and providing both psychological support (e.g., cognitive behavioral therapy) and initiating proper treatment (e.g., dilatators) [41]. Also, CRT-BT is not free of sexual dysfunction due to vaginal shortening, dryness, pain during intercourse, and compromised enjoyment [42].

We report acceptable surgical complication rates, considering the complication rates described for radical hysterectomy in early-stage cervical cancer. The EORTC-GCG performed a prospective, randomized trial of surgical drains versus none following radical hysterectomy [43]. Acute complications were seen in 128/234 entered patients (55%), and long-term complications were seen in 13%. Red blood cell transfusions were required in 32%. Here, we reported (albeit retrospectively) a much lower early complication rate (19.5%) and transfusion rate (8.3%).

Completion surgery is a valid method to ascertain the extent of RD in patients treated with CRT, which could be an important prognostic factor [10]. Identification of patients with RD after CRT is generally based on clinical examination, findings on MRI, and/or cervical biopsy results [44, 45]. Nine percent of our patients had RD after CRT-S of whom 26% grossly. Residual disease was seen in 44% of patients considered to have complete clinical and radiological responses. MRI evaluation after concomitant radiochemotherapy is insufficient to assess residual disease, with a reported sensitivity of 77.8% and specificity of 41.7% [46]. Residual tumor is more likely in AC because it is less radiosensitive compared to SCC [11]. In our cohort, the majority of AC patients (19 patients, 82.6%) showed a partial response on MRI and only two showed a pathologically complete response. Of note, a higher, but not statistically significant, 5-year OS was seen for AC compared to SCC: 79% and 71%, respectively. It is hypothesis generating that adenocarcinoma LACC may benefit from surgery.

Chemoradiation sterilized pelvic lymph node disease in more than 82%. All positive lymph nodes were boosted (simultaneously) up to 60 Gy, as is now recommended by the European Society of Gynaecological Oncology/European Society for Radiotherapy and Oncology/European Society of Pathology Guidelines [5]. Rouzier et al. found that residual pelvic lymph node involvement after radiation therapy alone was an independent predictive factor of local recurrence [47]. We could not confirm this: our 5-year LC is 88% for N1 status and 91% for N0 status at diagnosis (no significant difference).

The limitations of this study include its retrospective, single-institution design with limited sample size and possible selection bias and that clinical outcome analyses were largely descriptive in nature with no multivariable analyses being included. Of note, this study is unable to attribute causation due to the lack of a direct comparison standard treatment group. The strengths of this study are, besides its unique patient cohort allowing pathological response assessment after CRT, the long-term follow-up without patients who were lost to follow-up, rendering our findings solid and reliable.

## Conclusions

Completion surgery following CRT for LACC was safe and associated with durable LC and PC, which could arguably have contributed to a longer DFS and OS duration in stage III/IV and adenocarcinoma patients specifically. In other LACC patients, the survival outcome is comparable to historic data from CRT followed by brachytherapy, the current golden standard. Prospective, randomized studies are needed to corroborate our findings.

## Supplementary Information

**Additional file 1: **Survival outcomes. **Table S1.** Survival data. **Table S2.** univariate cox regression analysis of preoperative clinical and pathological features as prognostic factors for overall survival. **Table S3.** univariate cox regression analysis of preoperative clinical and pathological features as prognostic factors for disease free survival. **Fig. S1.** OS, DFS, PC and LC for the intention to treat group. **Fig. S2.** OS, DFS, PC and LC stratified according to FIGO 2009 (left) and FIGO 2018 (right).

## Data Availability

The datasets used and/or analyzed during the current study are available from the corresponding author upon reasonable request.

## Abbreviations

OS: Overall survival
LC: Local control
PC: Pelvic control
LACC: Locally advanced cervical cancer
CTCAE: Common Terminology Criteria of Adverse Events
^18^FDG PET-CT: Fluorine-18-fluorodeoxyglucose positron emission tomography
CRT: Concurrent chemoradiotherapy
SOC: Standard of care
RCT: Randomized controlled trial
CRT-S: Concurrent chemoradiotherapy followed by completion surgery
CRT-BT: Concurrent chemoradiotherapy followed by brachytherapy
IMRT: Intensity-modulated radiotherapy
FIGO: International Federation of Gynecology and Obstetrics
VMAT: Volumetric modulated arc therapy
MRI: Magnetic resonance imaging
ITT: Intention-to-treat
CTCAE: Common Terminology Criteria of Adverse Events
RD: Residual disease
AC: Adenocarcinoma
SCC: Squamous cell carcinoma
GI: Gastrointestinal
GU: Genitourinary
retroEMBRACE study: IntErnational MRI-guided BRAchytherapy in Cervical cancer study
