# Supplementary material for: Completion surgery after intensity-modulated arc therapy for locally advanced cervical cancer: long-term follow-up and update on surgical outcome and oncologic results of a unique tertiary care single-center retrospective cohort
Source: World J Surg Oncol. 2023 Mar 8;21:84. doi: 10.1186/s12957-023-02971-5 (PMC9993655; doi:10.1186/s12957-023-02971-5)

Supplemental data for the ITT group.

SURVIVAL OUTCOMES

**Overall survival (OS)**

The 5/10-year OS (95% CI) are 67% (66 – 82%) /49 % (37% - 66%) for the intention to treat group. When stratified according to histology, the 5-year OS is respectively 79% (95% CI 63-100) for adenocarcinoma and 65% (95% CI 56 – 75) for squamous cell carcinoma respectively. This difference was not significant.

**Disease free survival (DFS)**

The 5y and 10y DFS are 68% and 67%. Stratified by histology, the 5-year DFS (95% CI) is 61,2% (37,2% - 100%)/ 68,7% (60,1%-78,6%) respectively for adenocarcinoma/squamous cell carcinoma in the intention to treat group.

**Local control**

The 5y and 10y LC are both 93%. Stratified by histology, the 5-year LC (95% CI) is 95% (86 – 100) for adenocarcinoma and 88% (82-94) for squamous cell carcinoma.

**Pelvic control**

The 5-year pelvic control is 85%. When stratified according to histology, 5-year pelvic control (95% CI) does not differ significantly: 95%(86-100) for adenocarcinoma and 83% (76-91) for squamous cell carcinoma respectively.

+ supplemental table S1 (zie word document tables)

+ supplemental figure S1 (zie uitleg bij figures)

# Supplemental tables and Figures

Table S1: Survival data

|  | **Surgery group** | **Intention to treat** |
| --- | --- | --- |
| 5-year OS  Stratified by: | 72,5% (95% CI 64,2% - 81,8%) | 67,3% (95% CI 65,6 – 81,5%) |
| - Adenocarcinoma | 79,1% (95% CI62,6% - 100%) | 79,1% (95% CI 62,6-100) |
| - Squamous cell carcinoma | 70,9% (95% CI 61,7% - 81,5%) | 64,8% (95% CI 55,7 – 75,4) |
| - Node positive (N + ) | 70,4% (95% CI 58,5% - 84,7%) | 65,7% (95% CI 53,9% - 80,0%) |
| - Node negative (N -) | 74,5% (95% CI 63,6% - 87,3%) | 68,8% (95% CI 57,9% - 80,0%) |
| 10-year OS  Stratified by: | 53,1% (95% CI 40,1% - 70,3%) | 49,3% (95% CI 37,1% - 65,6%) |
| - Adenocarcinoma | 71,2% (95% CI 52,1%-97,4%) | 71,2% (95% CI 52,1% - 97,4%) |
| - Squamous cell carcinoma | 46,9% (95% CI 31,8%-69,1%) | 42,9% (95% CI 28,9% - 63,5%) |
| - Node positive (cN + ) | 48,1% (95% CI 28,8% - 80,2%) | 44,9% (95% CI 26,8% - 75,2%) |
| - Node negative (cN -) | 57,3% (95% CI 41,5% - 79,1%) | 53,0% (95% CI 38,1% - 73,7%) |
| 5-y DFS  Stratified by: | 73,6% (95% CI 65,4% - 82,8%) | 68,3% (95% CI 60,1%-77,6%) |
| - Adenocarcinoma | 61,2% (95% CI 37,2% - 100%) | 76,6% (95% CI 60,5% - 96,9%) |
| - Squamous cell carcinoma | 72,5% (95% CI 66,6% - 84,9%) | 68,7% (95% CI 60,1% - 78,6%) |
| - Node positive (cN+) | 74,5% (95% CI 63,8% - 87,0%) | 68,2% (95% CI 57,3% - 81,2%) |
| - Node negatief (cN-) | 73,7% (95% CI 62,4% - 87%) | 69,2% (95% CI 58,0%-82,6%) |
| 5y PC | 90,3% (95% CI 85,1%-95,9%) | 85,1% (95%CI 79,0% - 91,8%) |
| 5y-LC  Stratified by: | 92,8% (95% CI 88,2% - 97,8%) | 89,3% (95% CI 84,0% - 95,0%) |
| - Adenocarcinoma | 95% (95% CI 85,0% - 100%) | 95% (95%CI 85,9% - 100%) |
| - Squamous cell carcinoma | 92,3% (95% CI (95% 87,0% - 98,0%) | 88,1% (95% CI 81,9% - 94,7%) |
| - Node positive (cN+) | 91,3% (95% CI 84,2% - 98,9%) | 88,6% (95% CI 81,1% - 96,9%) |
| - Node negatief (cN-) | 94,4% (95% 85,5% - 100%) | 90,0% (95% CI 82,7 – 97,9%) |

Table S2: univariate cox regression analysis of preoperative clinical and pathological features as prognostic factors for overall survival

|  | Β | HR | CI 95% | p-value |
| --- | --- | --- | --- | --- |
| Age, weeks | 0,03 | 1,03 | 1,01 - 1,06 | 0,01 |
| FIGO stage 2009 | -0,22 | 0,69 | 0.27 – 2,36 | 0,69 |
| Tumor size, cm | 0,06 | 1,06 | 0,87 – 1,29 | 0,543 |
| Histology (SCC, AC) | -0,63 | 0,53 | 0,21 – 1,36 | 0,188 |
| Grade | 0,05 | 1,05 | 0,65 – 1,70 | 0,84 |
| Surgical approach | 0,32 | 1,37 | 0,63 – 2,97 | 0,426 |
| cN | -0,16 | 0,85 | 0,47 – 1,54 | 0,587 |

Table S3: univariate cox regression analysis of preoperative clinical and pathological features as prognostic factors for disease free survival

|  | Β | HR | CI 95% | p-value |
| --- | --- | --- | --- | --- |
| Age, weeks | 0,002 | 1,00 | 0,98 – 1,03 | 0,83 |
| FIGO stage 2009 | - 0,35 | 0,70 | 0,259 – 1,9 | 0,49 |
| Tumor size, cm | 0,065 | 1,07 | 0,87 – 1,31 | 0,53 |
| Histology (SCC, AC) | 0,15 | 1,17 | 0,51 – 2,66 | 0,715 |
| Grade | 0,123 | 1,13 | 0,54 – 2,35 | 0,74 |
| Surgical approach | 0,091 | 1,10 | 0,51 – 2,33 | 0,81 |
| cN | 0,19 | 1,21 | 0,63 – 2,30 | 0,57 |

**Supplemental figure 1** OS, DFS, PC and LC for the intention to treat group.


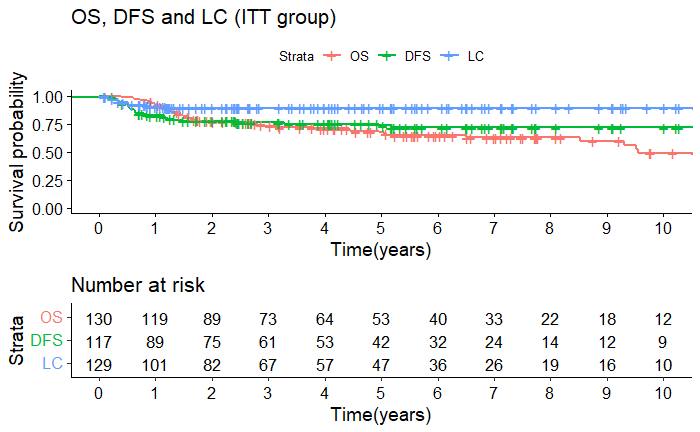


**Supplemental figure 2** OS, DFS, PC and LC stratified according to FIGO 2009 (left) and FIGO 2018 (right)


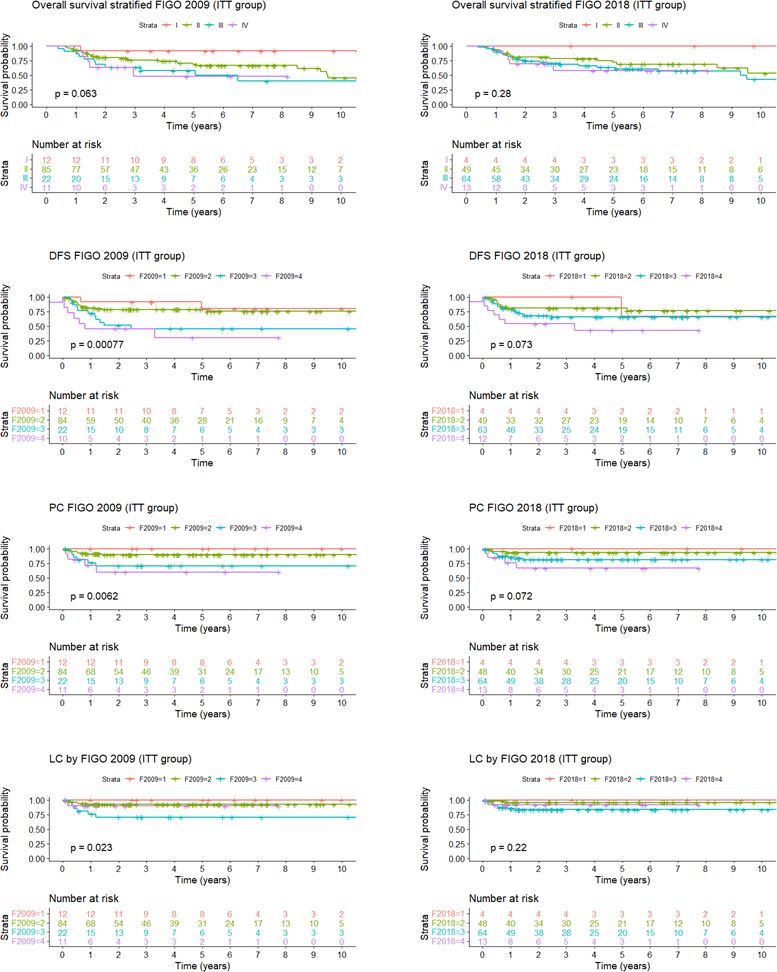

Supplement: Supplementary file 1 — Additional file 1: Survival outcomes. Table S1. Survival data. Table S2. univariate cox regression analysis of preoperative clinical and pathological features as prognostic factors for overall survival. Table S3. univariate cox regression analysis of preoperative clinical and pathological features as prognostic factors for disease free survival. Fig. S1. OS, DFS, PC and LC for the intention to treat group. Fig. S2. OS, DFS, PC and LC stratified according to FIGO 2009 (left) and FIGO 2018 (right). [file 12957_2023_2971_MOESM1_ESM.docx]
